# Dendritic cell immunotherapy advances for solid tumors: Vaccination and modulation

**DOI:** 10.1016/j.xcrm.2025.102412

**Published:** 2025-10-13

**Authors:** Georgia Clayton, Elisa C. Toffoli, Tanja D. de Gruijl, Yvette van Kooyk

**Affiliations:** 1 Department of Molecular Cell Biology and Immunology, Amsterdam UMC, Vrije Universiteit Amsterdam, 1081 HV Amsterdam, the Netherlands; 2 Cancer Immunology, Amsterdam Institute for Infection and Immunity, Amsterdam, the Netherlands; 3 Department of Medical Oncology, Amsterdam UMC, Vrije Universiteit Amsterdam, 1081 HV Amsterdam, the Netherlands; 4 Cancer Biology and Immunology, Cancer Center Amsterdam, Amsterdam, the Netherlands

**Keywords:** dendritic cells, tumor microenvironment, therapies, vaccination

## Abstract

Dendritic cells (DCs) are potent antigen-presenting cells, key for inducing anti-tumoral immune responses in the tumor microenvironment (TME) and tumor-draining lymph nodes. Within the TME, immunosuppressive signals often render DCs dysfunctional, hindering their propagation of T cell-mediated cancer cell death and tumor regression. DC-based immunotherapy has been investigated for over two decades, aiming to induce anti-tumor immunity by directly delivering DCs or antigens through vaccination or by enhancing the anti-tumor functions of existing DCs within the TME. Despite some progress, clinical benefit in many patients is still limited. As our understanding of the complex interactions that occur in the TME deepens and new technologies emerge, novel DC immunotherapy strategies are continuously being developed and advanced into clinical trials. This review provides an updated summary of the latest advances in these therapies, identifying trends that correlate with successful outcomes, as well as the challenges still being faced in the field.

## Graphical abstract

**Figure:**
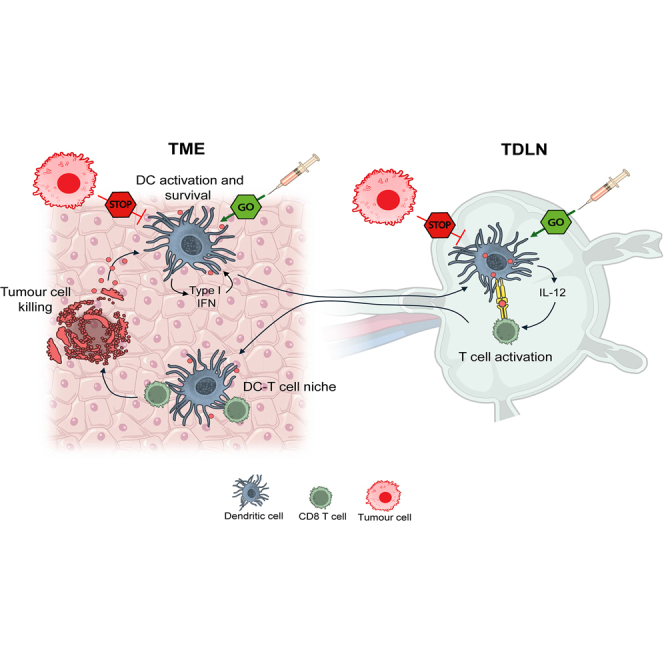

## Teaser

Dendritic cells (DCs) are crucial for anti-tumor immunity, yet the tumor microenvironment often impairs their function. Clayton et al. review advances in DC vaccines and modulation strategies, highlighting recent progress and clinical trends linked to improved therapeutic success.

## Introduction

### DC heterogeneity and function

Dendritic cells (DCs) are the principal antigen-presenting cells (APCs) of the immune system that bridge the gap between innate and adaptive immunity. They continuously sample their environment for antigens, which they phagocytose upon encounter, process into peptides, and present on their cell surface via major histocompatibility complex (MHC) molecules. During this process, DCs undergo maturation (evidenced by upregulation of CD80/83/86) and migration (associated with CCR7 expression, known as migratory DCs [migDCs]) to proximal draining lymph nodes (LNs) or tertiary lymphoid structures (TLSs) to prime naive T cells.^1^^,^^2^ Beyond antigen presentation, DCs secrete immunostimulatory cytokines upon engaging receptors such as CD40 and Toll-like receptors (TLRs). For example, CD40-CD40L crosstalk between DCs and T cells upregulates CD80/86 and the expression of interleukin (IL)-12 and interferons (IFNs).^3^ Similarly, activation of the stimulator of interferon genes (STING) pathway by DNA within DCs leads to type I IFN production and enhances DC maturation.^4^ DC-mediated cytokine production can enhance natural killer (NK) cell activity, an effector innate immune cell that can attack and eliminate target cells independent of prior immune sensitization,^5^ as well as attract effector T cells to the TME, creating niches where they can survive and thrive.^6^

DCs comprise a diverse population shaped by developmental origins and environmental cues. In mice, hematopoietic stem cells in the bone marrow can give rise to common myeloid and lymphoid progenitors. While both can give rise to DCs, under steady state, most are derived from the myeloid lineage.^7^ From here, differentiation can continue in the bone marrow to generate plasmacytoid DCs (pDCs). Alternatively, specialized precursors (pre-DCs) differentiate from progenitors and migrate to both lymphoid and non-lymphoid tissues, further differentiating into conventional DCs (cDCs), including cDC1 and cDC2, assisted by granulocyte-macrophage colony-stimulating factor (GM-CSF).^8^^,^^9^^,^^10^ Moreover, DCs can arise from tissue monocytes, forming monocyte-derived DCs (MoDCs), a major DC source during inflammation. pDCs circulate in the blood, are relatively abundant in LNs, and accumulate at inflammatory sites, contributing to the immune response.^11^ They specialize in viral sensing through TLR7/8/9 expression, producing type I and III IFN and cytokines that promote cDC1 maturation and NK cell function.^12^ cDC1 and cDC2 are characterized by their ability to phagocytose antigens and migrate to the LN, where they present antigens to naive T cells. In mice, cDC1s preferentially present peptide-MHC-I complexes to CD8 T cells, whereas cDC2s present peptide-MHC-II complexes to CD4 T cells, promoting Th1, Th2, and Th17 polarization. In human tissue, the subset nomenclature is maintained, but functional differences among cDC1, cDC2, and MoDCs are less clear due to their similar cross-presenting abilities.^12^^,^^13^^,^^14^ DC classification is under continuous refinement. For instance, it is now known that cDC2s encompass several functionally distinct states, while the newly described subset cDC3 shares characteristics with both cDC2s and MoDCs.^12^^,^^15^^,^^16^ Human DC subset ontogeny has mostly been investigated through *in vitro* differentiation models; while there is evidence in steady state that cDC subsets can originate from pre-DC lineages, the pDCs and DC3 differentiation pathways are less clear, with limited validation techniques available. Further details are discussed in a recent review on this topic.^17^

### Impaired functions of DCs in the tumor microenvironment

Cancer is a highly heterogeneous disease and the second leading cause of death worldwide. It is characterized by uncontrolled growth and spread of abnormal cells within a complex tumor microenvironment (TME) consisting of both immune and non-immune cells.^18^ DCs play crucial roles in the cancer-immunity cycle, which proposes that cancer immunity is a self-propagating cycle, from tumor antigen release, DC presentation to T cells in the tumor-draining lymph nodes (TDLNs), subsequent T cell migration into the TME, and T cell-mediated tumor killing.^6^^,^^19^ However, a hallmark of tumor progression and therapeutic resistance is the reprogramming of the immune system in favor of pro-tumoral immunity; therefore, DC function is hampered both in the TME and TDLN. DCs can have defective antigen uptake and processing, maturation, and LN migration, with detrimental effects on downstream immune responses, as evidenced by low DC counts, defective development, and impaired T cell priming in cancer patients.^20^ Skewing of the DC phenotype is driven by a complex interplay of factors, with key drivers being anti-inflammatory cytokines, predominantly secreted by tumor cells and cancer-associated fibroblasts, such as IL-6, IL-10, vascular endothelial growth factor (VEGF) and transforming growth factor (TGF)-β. IL-10 and TGF-β, for instance, inhibit DC maturation and antigen presentation by suppressing MHC and co-stimulatory molecule expression, generating tolerogenic DCs.^20^^,^^21^^,^^22^^,^^23^ The TME also suppresses DC activation signals (e.g., CD40L and TLR ligands), limiting inflammatory cytokine secretion.^24^ DC migration from the TME to TDLNs can be restricted by TGF-β and VEGF, as well as by physical barriers of the TME, such as dense extracellular matrix, to prevent priming of T cells.^25^ Furthermore, DC expression of the immune checkpoint protein, programmed death ligand 1 (PD-L1), can be upregulated by hypoxia and regulatory T cells (Tregs), which contributes to immunosuppression by limiting T cell activation in the TDLN.^24^^,^^26^^,^^27^ Similarly, hypersialylation in the TME promotes engagement with Siglec receptors on DCs to act as an immune checkpoint and induce tolerance.^28^^,^^29^^,^^30^

**Figure 1:**
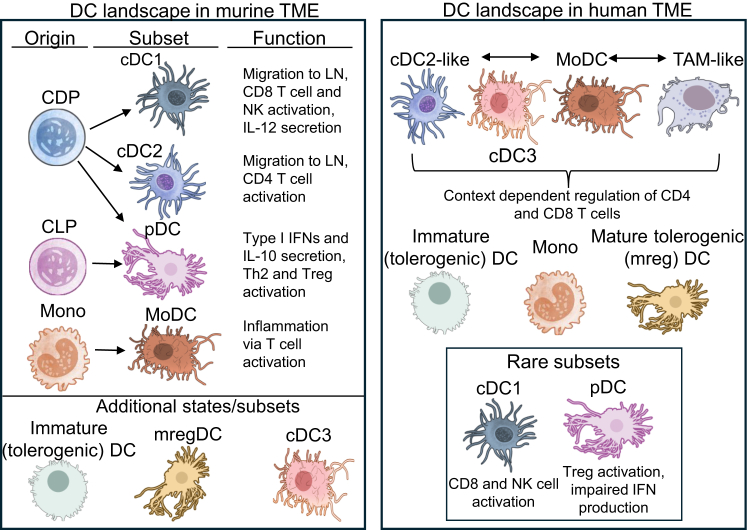
Dendritic cell subsets identified within the murine and human tumor microenvironment Lineage tracing in mice has allowed identification of CDP, CLP, and monos as precursors to DCs. In mice, cDC1s represent the major cross-priming subset, associated with improved anti-tumor responses. Although similar functions are observed in human tumors, their presence is extremely rare. In the human TME, CLEC10A cDC2-like cells are often reported as the most common DC subset, with CD88/89 expression indicating a monocyte origin, hence generating a spectrum of cDC2 to moDCs to TAMs, while in mice these subsets are more distinctly defined. Murine data suggest that cDC3s constitute a lineage independent of pDCs, cDCs, and monocytes, exhibiting characteristics that overlap with both cDC2s and monocytes, making their ontogeny and functional classification complex. mregDCs encompass a state of mature DCs, found in both the human and mice TME, that are characterized by their immunoregulation capabilities. Their role, along with pDCs in the TME, is still under investigation with both pro and anti-tumoral properties identified. Made using NIAID NIH BIOART (bioart.niaid.nih.gov/bioart). Abbreviations: cDC, conventional DC; CDP, common DC progenitor; CLP, common lymphoid progenitor; DC, dendritic cell; moDC, monocyte-derived DC; monos: monocytes; mregDCs: mature and regulatory DCs; pDC, plasmacytoid DC; TAMs, tumor-associated macrophages; TME, tumor microenvironment.

Within the TME, DC classification can become obscured due to their phenotypic plasticity and complex environmental cues.^15^^,^^16^ Characterizing DCs in the TME has been reviewed extensively elsewhere,^31^ but much remains to be understood. The bulk of our current understanding comes from murine tumor models and single-cell RNA sequencing or flow-cytometric analyses of patient-derived tumors. Based on CD88/CD89 expression confirmed by flow cytometry, most—if not all—mononuclear myeloid cells in the solid TME are monocyte derived. Monocytes recruited to the TME are influenced by prevailing immune suppressive cytokines, hence mostly adopting an immune suppressive macrophage-like appearance, but can also be driven to a more DC-like state (e.g., cDC2-like).^32^^,^^33^ Therefore, current evidence supports the existence of a cDC2-to-monocyte continuum.^16^^,^^34^^,^^35^ We and others have shown predominant roles of IL-6 and prostaglandins, derived from tumor-derived supernatants, or sialylation of tumor cells, in skewing monocyte differentiation away from a DC phenotype to an M2-macrophage-like state with pro-tumoral and immune suppressive features.^28^^,^^36^^,^^37^ Mature and regulatory DCs (mregDCs) expressing LAMP3 and PD-L1 represent a mature cDC state with immunosuppressive features. They have been found to have both pro- and anti-tumoral functions within solid TMEs.^38^^,^^39^ Figure 1 summarizes common DC subsets identified in the TME of mice and humans.

### Targeting DCs as a therapeutic avenue for cancer

**Figure 2:**
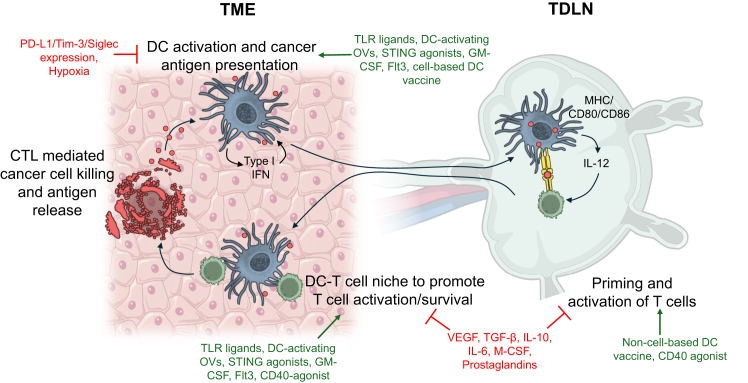
Suppressive factors inhibit the role of dendritic cells (DC) in the cancer-immunity cycle, which DC-based immunotherapies have the potential to overcome DC maturation and activation in the TME and antigen presentation in the TDLN are essential for tumor-specific CTL induction, attraction, activation, and survival to promote cancer cell killing. The functions of DCs in the cancer-immunity cycle can be hindered by immunosuppressive signals in the TME (highlighted in red). DC vaccination can introduce DCs to the TME or TDLN via cell-based therapies; alternatively, they can promote antigen presentation to T cells via non-cell-based approaches (e.g., DNA or mRNA vaccines). STING agonists, TLR ligands, and cytokines can activate DCs by stimulating their maturation and proliferation, promoting type I IFN secretion that can act in a positive feedback loop. CD40 agonists can bind to the CD40 receptor on DCs, licensing them to activate antigen-specific T cells through the upregulation of MHC and co-stimulatory molecules. Made using NIAID NIH BIOART (bioart.niaid.nih.gov/bioart). Abbreviations: CTL, cytotoxic T lymphocyte; DC, dendritic cell; STING, stimulator of interferon genes; TDLN, tumor-draining lymph node; TLR, Toll-like receptor; TME, tumor microenvironment.

Given their dysfunction playing a key role in successful tumor progression, DCs are prime targets for tumors to escape immunity, but also for immunotherapies to achieve tumor control (Figure 2). For effective immune defense in the TME and TDLN, DCs need to be not only present but also effectively activated and matured via environmental signals. DC-based immunotherapy can directly boost DC numbers or supply TME-lacking cytokines and growth factors needed to promote agonistic stimulatory activity and harness their role in the cancer-immunity cycle.^19^ DC-targeting therapies have been investigated since the 1990s^40^ and, since then, numerous novel approaches have emerged, aiming to improve upon the efficacy and tolerability of previous therapies.

Therapies targeting DCs also have the potential to benefit other immunotherapies that rely on T cell activation, such as immune checkpoint inhibitors (ICIs). ICIs have improved patient outlook and changed the standard of care (SoC) treatment for several tumor types (e.g., melanoma and lung cancer), including patients who exhibit resistance to standard therapy. Anti-cytotoxic T lymphocyte-associated protein (αCTLA-4), anti-programmed death 1 (αPD-1), αPD-L1, and anti-lymphocyte-activation gene 3 (αLAG-3) are currently registered for the treatment of solid tumors.^41^^,^^42^ However, due to the heterogeneity of the immune landscape, ICI success is limited to certain cancer types and patient subsets, specifically those with higher antigenicity and the pre-existence of an adequate anti-tumor immune response.^43^ DC-targeting combination therapy has the potential to expand the efficacy of ICIs by promoting a favorable TME with a high lymphocyte infiltration rate and T cell activation through DC-containing niches.^44^^,^^45^ This is supported by work in mice with *Batf3* knockout DCs, which showed that the anti-tumor effects of PD-1 inhibition required both *Batf3*-dependent DCs cross-presenting antigens to T cells to elicit antigen-specific immunogenic responses and effector T cell attraction to the TME.^46^ Indeed, functional DC infiltration has been shown to correlate with immunotherapy success in cancer patients,^47^ and DC therapy with ICI combination is being actively investigated in the clinic.^44^^,^^48^

This review highlights the clinical progress in the last 5 years of DC-based immunotherapies that we categorize into anti-tumoral therapeutic vaccination approaches, which directly provide antigens primarily, but not necessarily exclusively, to DCs,^49^ and immunomodulatory therapies that can enhance the anti-tumor functionality of DCs.

## Methods of DC targeting—Developments in monotherapy and combination therapy approaches

### Anti-tumoral therapeutic vaccination

Anti-tumoral vaccination strategies focus on delivering specific antigens to DCs by loading DCs *ex vivo* or directly in patients. The former method commonly uses autologous DCs either directly harvested from blood, *in vitro* differentiated DCs from monocytes, or CD34^+^ progenitors. After differentiation and/or activation (as needed), these cells can be loaded with specific antigens (e.g., human epidermal growth factor receptor 2 [HER2] and melanoma antigen recognized by T cells [MART-1]) or, more commonly, co-cultured with tumor lysate (TL) to load a range of autologous tumor antigens. DCs can also be pulsed with neoantigen peptides derived from whole-exome and RNA sequencing of fresh biopsy tissue.^50^ Loading DCs directly into patients consists of loading specific antigens directly *in vivo* by various routes of administration (intravenous (i.v), intradermal, intramuscular, subcutaneous, or intracutaneous) and delivery methods (e.g., DNA; RNA; viral vectors; or synthetic long peptides, naked or formulated as liposomal nanoparticles). Targeted antigens can consist of shared tumor antigens (both “self” and “non-self” as virus-derived) or neoantigens, antigens arising from specific tumor mutations, or post-translational modifications allowing for a personalized approach for therapeutic cancer vaccines, as recently reviewed by Saxena and colleagues.^1^

Conventional *in vivo* antigen delivery lacks DC specificity, as antigens can also be (preferentially) internalized by other APCs, such as macrophages.^49^ Given that DCs are the most potent APCs and represent the preferred target for vaccination, several strategies have been developed to enhance the selectivity of antigen delivery to DCs. One approach is the use of antibodies targeting DC-related receptors (i.e., DEC205, CD40, or CLEC9A) fused to a specific antigen of choice.^49^ While extensive *in vitro* and *in vivo* studies have been performed, clinical progression of these antibodies has been limited, with only phase 1 clinical trials showing the safety of this approach without evidence of efficacy.^49^^,^^51^^,^^52^ Another strategy to increase delivery selectivity of antigens uses nanoparticles that can simultaneously deliver both an antigen, in the form of a peptide or mRNA, and immune adjuvants to DCs.^53^^,^^54^ Their DC selectivity can be further enhanced by introducing surface modifications to target DC membrane receptors, such as C-type lectin receptors (e.g., CD206, DEC205, DC-SIGN, and CLEC9A^53^^,^^55^); however, this field is still in early research stages.^55^ In contrast, non-DC-targeting mRNA lipid nanoparticle vaccines have been widely studied and approved for use during the COVID-19 pandemic, with multiple clinical trials analyzing their effects against malignancies yielding promising results, as recently reviewed by Sayour and colleagues.^54^

Currently, Sipuleucel-T is the only therapeutic anti-cancer vaccine approved for clinical use as treatment for metastatic castration-resistant prostate cancer. Sipuleucel-T is an autologous cellular vaccine consisting of peripheral blood mononuclear cells (PBMCs) *ex vivo* stimulated with PA2024, a fusion protein containing the prostatic cancer antigen, prostatic acid phosphatase, and the DC-activating cytokine GM-CSF. This vaccine has displayed limited efficacy, with a 4 month overall survival (OS) benefit without disease-free survival (DFS) improvements^56^; hence, there is large scope for improvement of anti-tumor vaccination strategies. Current elements that seem to characterize successful strategies for clinical advancement are one (or multiple) of the following characteristics: (1) the presence of an effective DC-activating agonist, (2) low tumor burden or early disease stage, (3) (controversially) a cold TME at baseline (i.e., lacking T cell infiltration), (4) well-timed combination therapies, and (5) measurable induction or boosting of anti-tumor T cell responses. A detailed overview of studies using anti-tumoral DC vaccination can be found in Table 1, including tumor type, delivery method, clinical outcomes, and immune monitoring information.

**Table 1:** Clinical studies on anti-tumoral therapeutic vaccination approaches

Therapy and reference	Reference	Delivery^a^	Tumor type	Tumor stage	Trial phase	*N*	Adverse events (*n* ≥ grade 3)	Clinical outcome	Immune monitoring highlights
4 arms: A. aMoDC stimulated with TL-loaded YCWPs with G-CSF, B. without G-CSF, C. placebo, D. placebo with G-CSF	Adams et al., 2023^57^	ID	MEL	III-IV	2b	144	not stated	36-month DFS: arm A. 55.8 vs. B. 24.4, C + D. 30.0% 36-month OS: arm A. 94.2, B 69.8, C + D. 70.9%	arm A compared to arm B: upregulation of genes associated with DC maturation and downregulation of genes related to DC suppression or immaturity.
4 arms: A. aMoDC stimulated with TL-loaded YCWPs with G-CSF, B. without G-CSF, C. direct administration of TL-loaded YCWPs, D. placebo	Carpenter et al., 2023^58^	ID	MEL	III-IV	2b	187	arm B: 1 arm D: 1	DFS: arm A. 55.4%, B. 22.9%, C. 60.9%, D. 27.2%. OS: arm A. 93.6%, B. 57.7%, C. 94.6%, D. 62.5% median follow-up: 35.8 months	–
aMoDC stimulated with TL-loaded YCWPs vs. control (aMoDC + unloaded YCWPs)	Vreeland et al., 2021^59^	ID	MEL	III-IV	2b	144	12 (8 in control group)	24-month DFS: 62.9% treatment vs. 34.8% control	–
3 arms: A. aMoDC loaded with TL only B. in combination with either Poly IC (TLR3) C. in combination with resiquimod (TLR7/8)	Everson et al., 2024^60^	ID	glioma	III-IV	2	23	0	mPFS in months: arm A. 5.5, B. 31.4, C. 8.1. mOS in months: arm A. 7.7, B. 52.5, C. 16.7	higher IFN type II response is associated with better clinical outcome. both TLR ligands induced (1) enrichment of both type I and II IFN downstream genes with a more heterogeneous and weaker response in the resiquimod group, (2) higher levels of blood Ki67^+^CD14^+^ classical monocytes, (3) reduced T cell exhaustion makers (CD38 and CD39) and increased PD-1 expression.
aMoDC loaded with allogeneic TL from PheraLys (mesothelioma cell line, MesoPher) after cytoreductive surgery and hyperthermic intraperitoneal chemotherapy	Dietz et al., 2023^61^	ID	MESO	IV	2	16	0	mPFS: 12 months mOS: not reached	MesoPher led to increases in CD8^+^ TEMRA cells, which positively correlated with PFS. A slight decrease in classical and non-classical monocytes was observed after one or three vaccinations, respectively.
aMoDC loaded with allogeneic TL obtained from PheraLys (mesothelioma cell line, MesoPher) vs. control	Aerts et al., 2024^62^	i.v. + ID	MESO	IA-IV	2/3	176	MesoPher: 19 vs. controls: 15	MesoPher led to no benefit in OS	MesoPher led to increases in proliferation of central memory T cells and CD4^+^ T cells, with concurrent increases in ICOS. These results correlated with PFS.
DNA VB10.16 (HPV) vaccine	Hillemanns et al., 2022^63^	IM	CIN	2–3	1/2a	34	1	94% reductions in lesion size with 10 CR. HPV clearance in 8/17 subjects	correlation between IFNγ T cell responses and lesion size reduction.
Th17-inducing aMoDC loaded with FRα peptides	Block et al., 2020^64^	ID	OC	IIIC-IV	1	19	0	mPFS: 12.1 months mOS: not reached median follow-up: 49.2 months	IFNγ^+^ T cell levels, antibody responses, and *in vitro* antibody-dependent cell cytotoxicity were higher in recurrence-free patients.
aDC (from CD34 progenitors) pulsed with the cytomegalovirus pp65 RNA protein with either tetanus-diphtheria (TD) or GM-CSF as adjuvant. 3 clinical trials combined	Batich et al., 2020^65^	ID	GBM	IV	1/2	23	not stated	5-year PFS: 33% in TD cohort and 36% in GM-CSF cohort mOS: TD: cohort 37.7 months and GM-CSF cohort 38.3 months	the TD cohort showed increased DC migration into draining LNs. Data not available for GM-CSF cohort.
aMoDC loaded with TL vs. placebo controls	Liau et al., 2023^66^	ID	GBM	IV	3	331	4	newly diagnosed GBM: mOS 19.3 vs. 16.5 control recurrent GBM: mOS 13.2 vs. 7.8 control	–
WT1 peptide-pulsed aDC. The vaccine was administered after one cycle of nab-paclitaxel plus gemcitabine	Koido et al., 2024^67^	ID	PDAC	III-IV	1	10	22	all patients responded: 7/10 PR + 3/10 long-term SD	long-term WT1-delayed-type hypersensitivity positivity was correlated to clinical outcome (mOS and mPFS not reached vs. 1.37 and 2.56 years for short-term responders). The long-term responders had higher levels of IFNγ or TNF production by CD4^+^ or CD8^+^ T cells as well as lower levels of Tregs and MDSCs.
Individualized neoantigen vaccine based on uridine mRNA-lipoplex nanoparticle (Cevumeran) in combination with atelizumab (αPD-L1)	Rojas et al., 2023^68^	i.v.	PDAC	I-III	1	16	1	mOS and mPFS not reached median follow-up: 18 months	patients with vaccine-expanded T cells (8/16) had a longer mPFS compared to patients without such responses (mPFS not reached vs. 13.4 months). no differences in the peripheral levels of DCs or monocytes were observed between responders and non-responders.
DCVAC: aMoDC loaded with antigens from EOC cell lines (OV-90 and SK-OV-3) parallel (Arm A) or sequential (Arm B) to chemotherapy vs. chemotherapy only controls (Arm C)	Rob et al., 2022^69^ + immune monitoring in Fucikova et al., 2022^70^	i.v.	OC	III	2	99	arm A: 28 arm B: 37 arm C: 31	2-year PFS: arm A. 47%, B. 75%, C. 46% mPFS: arm A. 20.2 months, B. not reached, C. 21.4 months 4-year OS: arm A. 71%, B. 79%, C. 63% mOS not reached for all arms	higher tumor mutational burden and CD8^+^ T cell infiltration were associated to clinical benefit in patients receiving chemotherapy while superior responses to DCVAC were observed with lower-than-median-tumor mutational burden and scarce CD8^+^ T cell infiltration independently from the chemotherapy timing.
Autologous monocyte-derived type-1-polarized DC vaccine (αDC1) loaded with HLA-A2 presented peptides derived from non-mutated tumor blood vessel antigens with oral dasatinib either in week 1 (arm A) or in week 2 (arm B)	Storkus et al., 2021^71^	ID	MEL	II-IV	2	13	0	ORR: arm A. 66.7% vs. B. 0% mPFS: arm A. 7.87 vs. B. 1.96 months mOS: arm A. 15.45 vs. B. 3.47 months	patient T cell response to vaccine and total (also non-vaccine) peptides determined in IFNγ ELISPOT was predictive of extended OS. Higher levels of OX40L were observed on αDC1 from non-responders. No differences in other DC markers, IL-12p70 or IL-10 levels were found.
TILs alone (arm A) or combined with aMoDCs loaded with NY-ESO-1 (arm B)	Lovgren et al., 2020^72^	ID	MEL (ICI resistant)	III-IV	1	10	multiple, but none associated with DC vaccination	all 4/10 patients in arm 2 had an objective response: 2 CRs, 1 long-lasting PR, and 1 short-term PR	arm B: no responses to delayed-type hypersensitivity testing against TL and NY-ESO-1 peptide.
TILs with MART-1 reactivity alone (Arm A) or combined with MART-1-loaded aMoDC (Arm B)	Saberian et al., 2021^73^	i.v.	MEL	III-IV	2	18	multiple, but none associated with DC vaccination	objective response: 30% (3/10) TILs only and 50% (4/8) combination. The study was not powered for differences in efficacy	higher levels of CD8^+^ cells in the TIL product were correlated with better response.
Vaccine targeting IDO and PD-L1 peptides (IO102-IO103) in combination with nivolumab (αPD-1)	Kjeldsen et al., 2022^74^	s.c.	MEL	Unres-IV	1/2	30	4	ORR: 80%; CR: 43%; PR: 37% mPFS: 26 months for all patients. Not reached by responders mOS: not reached	increases in PD-L1- or IDO-specific T cell responses in 93% of patients.
Autologous CD1c^+^ cDC and pDCs loaded with tumor peptides (arm A) or placebo (arm B)	Bol et al., 2024^75^	IN	MEL	IIIB/C	3	148	arm A: 5% arm B: 6%	2-year PFS: arm A 36.8% vs. B 46.9%. mPFS: arm A 12.7 vs. B 19.9 months mOS: not reached for both arms	functional antigen-specific T cell responses could be detected in 67.1% of patients tested in arm A vs. 3.8% in arm B.
aMoDC exposed to LNCaP (PrC cell line) (arm A) vs. placebo (arm B)	Vogelzang et al., 2022^76^	s.c.	PrC	IV	3	1182	arm A: 50.1%, arm B: 57.0%	OS: arm A. 23.9 vs. B. 24.3 months	–

(m)OS, (median) overall survival; (m)PFS, (median) progression-free survival; aDC, autologous DC; aMoDC: autologous monocyte-derived DC; CAR-T, chimeric antigen receptor T cells; cDC, conventional DC; CIN, cervical intraepithelial neoplasia; CLDN6, claudin 6; CR, complete response; DC, dendritic cell; DFS, disease-free survival; EOC, epithelial ovarian cancer; FRα, folate receptor alpha; GBM, glioblastoma; G-CSF, granulocyte colony-stimulating factor; GM-CSF, granulocyte monocyte colony-stimulating factor; HCC, hepatocellular carcinoma; HPV, human papillomavirus; ID, intradermal; IDO, indoleamine 2,3-dioxygenase; IFN, interferon; IL, interleukin; IN, intranodal; i.v., intravenous; LN, lymph node; MART-1, melanoma antigen recognized by T cells 1; MDSCs, myeloid-derived suppressor cells; MEL, melanoma; MESO, mesothelioma; OC, ovarian cancer; ORR, objective response rate; PD-(L)1, programmed death-(ligand)1; PDAC, pancreas ductal adenocarcinoma; pDC, plasmacytoid DC; Poly IC, polyinosinic:polycytidylic acid; PR, partial response; PrC, prostate cancer; s.c., subcutaneous; TD, tetanus-diphtheria; TEMRA, terminally differentiated effector memory; Th, T helper; TILs, tumor-infiltrating lymphocytes; TL, tumor lysate; TLR, Toll-like receptor; TNF, tumor necrosis factor; Unres, unresectable; WT1, Wilms’ tumor 1; YCWPs, yeast cell wall particles.

a of anti-tumoral therapeutic vaccine.

### Effective agonists

An example of a successful agonist is constituted by a vaccine combining TL-loaded in immunity-stimulating yeast cell wall particles (YCWPs), either pulsed *ex vivo* on autologous DCs or delivered as *in vivo* vaccination. YCWPs allow for both the delivery of TL to DCs and agonistic stimulation of innate immunity.^59^ In melanoma patients, both DFS and OS were prolonged.^57^^,^^58^^,^^59^ Interestingly, combination with granulocyte colony-stimulating factor (G-CSF) annulled the therapeutic effect by the maintenance of an immature DC phenotype.^57^^,^^58^ The administration of *ex vivo* loaded DCs was also compared to the *in vivo* delivery of TL with YCWPs, which showed similar clinical results, with evident advantages of the *in vivo* approach from both manufacturing and commercial perspectives.^58^ The presence of mature DCs and YCWPs without TL was found insufficient to induce a clinical response, suggesting that both YCWPs and TL are required for vaccination efficacy.^58^ Adding appropriate TLR stimulation can also boost vaccination efficacy. For instance, a recent phase 2 clinical trial in patients with grade III and IV glioma showed that combining an autologous TL pulsed DC vaccine with polyinosinic:polycytidylic acid (Poly:IC; TLR3) enhanced progression-free survival (PFS) and OS more efficiently than the addition of resiquimod (TLR7/8).^60^ Interestingly, while both TLR ligands induced type I and II IFN downstream genes, Poly:IC showed a stronger and more consistent response, with improved clinical outcomes correlating with higher IFN type II responses.

### Low tumor burden/early disease stage

A notable success in early-stage cancer is a phase 1/2 clinical study administering DNA-based human papillomavirus (HPV) vaccination to patients with cervical intraepithelial neoplasia. Complete response (CR) was achieved in 10/17 patients, with strong IFNγ responses correlating with tumor shrinkage.^63^ Similarly, a trial analyzing the effects of a T helper (Th)17-inducing autologous DC vaccine targeting folate alpha receptor (FRα) in patients with ovarian cancer (OC) in remission after SoC showed a favorable PFS, which correlated with the IFNγ response.^64^ Another example of the importance of a lower tumor burden is offered by trials analyzing MesoPher, a vaccine therapy consisting of *ex vivo*-generated MoDCs loaded with allogeneic lysate from a mesothelioma cell line.^61^^,^^62^ Interestingly, a phase 3 trial analyzing MesoPher combined with best supportive care had no effects on OS in patients with advanced mesothelioma,^62^ whereas administration after surgical debulking had promising clinical effects, which correlated with increased rates of CD8 terminally differentiated effector memory T cells.^61^

### Immune-cold tumors

Glioblastoma is an immune-cold tumor (lacks significant immune cell infiltration and activity) situated in a so-called “immune privileged” site, i.e., the brain. Multiple phase 1 clinical trials have tested a vaccination strategy consisting of autologous DCs differentiated from CD34^+^ progenitors pulsed with a cytomegalovirus (CMV)-specific antigen (pp65) and administered after SoC. In the context of reduced tumor burden, this strategy showed encouraging results, with one trial finding a 5 year DFS of 33% and another with a 5 year OS of 36%.^65^ Similarly, a clinically meaningful benefit was observed in a phase 3 clinical trial analyzing the effects of an autologous MoDC-based vaccination loaded with TL in combination with SoC in glioblastoma patients.^66^ A phase 2 clinical trial testing the effects of DCVAC, an autologous DCs vaccine loaded with antigens derived from OC cell lines (OV-90 and SK-OV-3), in OC patients showed that immune-cold tumors responded better to DCVAC than hot tumors, which responded better to chemotherapy.^70^ Furthermore, in pancreatic ductal adenocarcinoma (PDAC), which is classically characterized by an immune-cold TME, clinical outcomes appear linked to induced T cell responses. For instance, in patients with PDAC treated with a Wilms’ tumor 1 (WT1) peptide-pulse DC vaccine, sustained peptide-specific T cell responses were observed in long-term responders but not in short-term responders.^67^ Similarly, a phase 1 clinical trial analyzing the effects of cevumeran, an individualized neoantigen vaccine based on uridine mRNA-lipoplex nanoparticles, in combination with atelizumab (αPD-L1) in patients with PDAC showed that patients with vaccine-expanded T cells experienced significantly longer median PFS than those without such responses.^68^

### Immunotherapy combinations and the importance of timing of therapy

Anti-tumoral DC vaccination strategies have been combined with chemotherapy, targeted therapies, and ICIs, as well as more experimental approaches such as tumor-infiltrating lymphocyte (TIL) transfer. Key components of an effective combination strategy are the timing and sequencing of therapies, exemplified in a phase 2 clinical trial for OC, where DCVAC administration before chemotherapy (post-cytoreduction surgery) significantly improved PFS, unlike parallel administration.^69^ Similarly, in a phase 2 trial in patients with ICI-refractory advanced melanoma, simultaneous administration of dasatinib and an autologous monocyte-derived type-1-polarized DC vaccine (αDC1) loaded with non-mutated tumor blood vessel antigens (TBVAs) remarkably outperformed delayed dasatinib administration, with an overall response rate (ORR) of 66.7% vs. 0% and a mOS of 15.45 vs. 3.47 months.^71^

Promising combination therapy strategies involve leveraging the ability of DCs to stimulate T cells and other immune effector cells by combining DC vaccination with adoptive effector cell transfer or with approaches able to unleash endogenous effector cells, such as ICIs. Pilot studies analyzing the effects of anti-tumoral therapeutic vaccines combined with TILs show signs of clinical efficacy. In particular, combining TIL transfer with an NY-ESO-1-loaded MoDC vaccine in patients with ICI-resistant melanoma showed a response in 4/8 treated patients, with two long-lasting CR and one partial response (PR).^72^ Similarly, higher objective response rates were observed in patients with advanced melanoma treated with TILs combined with a MART-1 pulsed-DC vaccine compared to TIL-only controls (50% vs. 30%).^73^ However, as both studies included a limited number of patients, larger studies are needed to confirm these results.

Anti-tumoral therapeutic vaccination has the potential to augment ICI efficacy by inducing intratumoral inflammation and responses against predicted neoantigens. Although patients with metastatic melanoma show great sensitivity to αPD-1 therapy, 50% of patients progress due to resistance. A phase 1/2 clinical trial analyzing the combination of a peptide-based vaccine against indoleamine-pyrrole 2,3-dioxygenase (IDO) and PD-L1 combined with nivolumab (αPD-1) showed promising results with an ORR of 80% and 43% CRs. The median PFS was 26 months, and the mOS was not reached. Vaccine-specific responses were observed in more than 93% of patients.^74^ Although this trial did not include an ICI-only arm, both ORR and median PFS were higher than those observed for nivolumab monotherapy in the CheckMate 067 study, suggesting the combination of ICIs with DC-mediated T cell stimulation has the potential to enhance ICI efficacy.

### Contradictory results

While the aforementioned characteristics seem to correlate with good clinical results, this is not always the case. A phase 3 clinical trial conducted in patients with resected stage IIIB/C melanoma showed no clinical benefit after treatment with autologous DCs (cDC2 and pDC) loaded with tumor antigens, despite the detection of antigen-specific T cell responses in 67.1% of patients and the reduced tumor burden in the adjuvant setting.^75^ Moreover, a phase 3 clinical trial analyzing the effects of autologous DCs exposed to LNCaP (a prostate adenocarcinoma cell line) in patients with castration-resistant prostate cancer, characterized by an immune desert-like phenotype, showed no OS benefits compared to chemotherapy and prednisolone treated controls.^76^ However, the administration of prednisolone during vaccination may have hampered vaccine-mediated immune effects.^77^ In conclusion, in the right setting, anti-tumoral vaccination has the potential to induce an effective anti-cancer response. Future studies should focus on further unraveling the key mechanisms underlying this response.

## Immunomodulatory therapies that enhance the anti-tumor functionality of DCs

Immunomodulatory strategies focus on driving the activation of DCs already present within the TME (and TDLN) without *ex vivo* antigen loading to stimulate their secretion of pro-inflammatory cytokines, migration to the TDLN, and subsequent antigen presentation to boost anti-tumor T cells. As with most TME modulatory therapies, these approaches are not DC specific but can influence an array of cell types and signaling pathways in the TME. Immunomodulatory therapies that alter DC function undergoing active exploration include CD40 agonists, immune-stimulating cytokines, STING agonists, and TLR ligands. Moreover, DCs within the TME can be stimulated by specific (oncolytic) viruses. Details of recent clinical trials utilizing DC immunomodulatory agents, including details of tumor type, delivery method, clinical outcomes, and immune monitoring information, are presented in Table 2.

**Table 2:** Clinical studies using DC immunomodulatory approaches

Therapy	Reference	Delivery^a^	Tumor type	Tumor stage	Trial phase	*N*	Adverse Events (*n* ≥ grade 3)^b^	Clinical outcome	Immune monitoring highlights
**CD40 agonists**									

RO7300490: bispecific antibody targeting CD40 and fibroblast activation protein	Melero et al., 2022^78^	IV	various	ADV	1	296	2	14/26 SD	treatment resulted in increased DC-LAMP^+^ DCs within the tumor and a reduction of CLEC9A^+^ DCs as well as an increase in intratumoral B cells and formation of pre-TLS.
Selicrelumab (agonistic CD40) monotherapy (Arm A) or combined with chemotherapy (gemcitabine and nab-paclitaxel) (arm B)	Byrne et al., 2021^79^	IV	PDAC	RES	1	15	8	mPFS: Arm A. 9.8 months vs. B. not reached mOS: arm A. 23.4 months vs. B. not reached 1 year OS: 100% for both arms	selicrelumab treatment led to immune-enriched tumors, with 82% showing T cell infiltration compared to 37% in untreated and 23% in chemo/radiotherapy-treated tumors. These patients also exhibited higher IT mature DC levels, fewer IT M2 macrophages, and elevated serum inflammatory cytokines (CXCL10 and CCL22) post-treatment.
Mitazalimab (αCD40) combined with mFOLFRINOX (fluorouracil, leucovorin, oxaliplatin, and irinotecan)	Van Leathem et al., 2024^80^	IV	PDAC	IV	1b/2	70	55	1 CR, 22 PR, 22 SD ORR: 40% disease control rate: 79% mPFS: 7.7 months mOS: 14.3 months After 18 months, the median OS was 14.3 months (42.1% response rate)	at day 8 of cycle 1, increased levels of CD38^+^ NKT cells were observed compared to baseline, with abundance significantly correlated with best depth of response.

**STING agonists**									

STING agonist (MK-1454) as monotherapy (arm A) or in combination with pembrolizumab (arm B)	Harrington et al., 2018^81^	IT	various	ADV	1	25	arm A: 9% arm B: 14%	arm A: 24% PRs were seen with reductions in both target injected and non-injected lesions (median −83%). DCR: 20% arm B: no CR/PR. DCR: 48%	in both arms elevations in serum cytokines IL-6 and IL-10 and STING-induced gene expression were observed in blood.
STING agonist (MIW815) and spartalizumab	Meric-Bernstam et al., 2023^82^	IT	various	ADV-IV	1b	106	14	progressive disease occurred in 56 patients 1 CR, 10 PR, 20 SD. ORR: 10.4%	dose-dependent increase of IFNβ and responders had increase CD8^+^ T cells.
SYNB1891, an Engineered *E. coli* Nissle strain expressing STING agonist, with (Arm A) and without atezolizumab (arm B)	Luke et al., 2023^83^	IT	various	III-IV	1	34 (24 arm B)	31%	9/25 evaluable patients reached SD (of which 6/9 in arm B) SD in 4 participants refractory to prior PD-1/L1 antibodies	upregulation of IFN-stimulated genes, chemokines/cytokines, and T cell response genes in tumor biopsy compared to baseline.

**Cytokines**									

GS-3583 Flt3 agonist Fc fusion protein	Tolcher et al., 2024^84^	IV	various	ADV	1b	13	7 (with 1 grade 5)	5 SD	GS-3583 exposure led to expansion of cDC1 and cDC2 at all dose levels, as well as increases in monocyte’s levels.
anti-DEC-205-NY-ESO-1 vaccine and polyinosinic:polycytidylic acid, with (Arm A) or without (Arm B) prior Flt3 ligand (CDX-301) treatment (Arm B)	Bhardwaj et al., 2020.^85^	s.c.	melanoma	resected	2	60 (30 in each arm)	arm A: 5 arm B: 8	clinical benefit not assessed, but it was noted that, in arm A, 13 participants had disease progression and, in arm B, 9 had progressed	treatment with Flt3L ligand (arm A) increased cDCs, pDCs, monocytes, NK cells, CD8 memory T cells and Tregs, compared to arm B.
GM-CSF- secreting whole-cell pancreatic cancer vaccine (GVAX) with low-dose cyclophosphamide alone (arm A), with αPD-1 antibody nivolumab (arm B), and with both nivolumab and αCD137 agonist antibody urelumab (arm C)	Huemann et al., 2023^86^	ID	PDAC	RES	platform trial	arm A: 17 arm B: 18 arm c: 11	Arm C: 1	arm A: DFS: 13.9, OS: 23.6 arm B: DFS: 15, OS: 27 arm C: DFS: 33.5, OS: 33.6	CD8 T cells increased in arm B compared to arm A but did not further increase in arm C; Tregs significantly increased in arm C.
GM-CSF in combination with the αGD2 monoclonal antibody naxitamab	Mora et al., 2025^87^	s.c.	neuroblastoma	III-IV	2	74	158	52 patients evaluable for efficacy ORR: 50%. 20 CR and 6 PR. 1-year PFS: 35% 1-year OS: 93%	N/A

**Oncolytic viruses**									

T-VEC	Ressler et al., 2025^88^	IT	BCC	difficult to resect	2	18	0	6 CR, 4 PR, 8 SD. ORR: 55.6% pathological CR rate: 33.3% 6-month OS: 100%	responders had IT increases in cytotoxic T cells, B cells, and myeloid cells, as well as reductions in Tregs. No difference in DC abundance between patients with a pathological CR or non-complete response.
T-VEC in combination with ipilimumab (αCTLA-4) (Arm A) vs. ipilimumab monotherapy (Arm B)	Chesney et al., 2023^89^	IT	MEL	IIIB-IV	2	198	arm A: 44, arm B: 41	ORR: arm A. 35.7% vs. B. 16.0% durable response rate: arm A. 33.7% vs. B. 13.0% mPFS: arm A. 13.5 vs. B. 6.4 months estimated 5-year OS: 54.7% vs. 48.4% follow-up: arm A. 49.4 vs. B. 35.8 months	N/A
T-VEC in combination with pembrolizumab (αPD-1) (Arm A) vs. pembrolizumab monotherapy (Arm B)	Chesney et al., 2023^90^	IT	MEL	IIIB-IV	3	692	arm A: 70 arm B: 54	ORR: arm A. 48.6% vs. B. 41.3% durable response rate: arm A. 42.2% vs. B. 34.1%	N/A
T-VEC in combination with pembrolizumab (αPD-1). Four arms based on disease: unresectable/metastatic MEL with primary (arm A) or secondary (arm B) resistance to ICIs. Resectable MEL with PFS < 6 months after ICI (arm C) or > 6 months (arm D)	Robert et al., 2024^91^	IT	MEL	IIIb-IV	2	71	26	ORR: arm A. 0%, B. 6.7%, C. 40%, D. 46.7% mPFS: arm A. 5.5 and B. 8.2 months. Not estimable for arm C and D	N/A
T-VEC in combination with pembrolizumab (αPD-1)	Harrington et al., 2020^92^	IT	HNSCC	R-IV	1b	36	arm A: 5 with 1 grade 5 arm B: 6	5 PR. 10 patients not evaluable due to early death. mPFS: 3 months mOS: 5.8 months	N/A
T-VEC monotherapy or combined with pembrolizumab (αPD-1). three arms: A. non-HCC liver metastasis treated with T-VEC only (arm A) or the combination (arm B). HCC treated with the combination (arm C)	Hecht et al., 2025^93^	IT or intrahepatic	various	IV	1b/2	74	3	ORR: arm A 0%, B 8.3%, C 13.6% mPFS: arm B 2 vs. C 8.1 months mOS: arm B 7.8 vs. C 12.8 months triple-negative BrC sub-analysis: ORR: 16.7% mPFS: 2.9 months, mOS: 10.2 months	N/A
AdV DNX-2401 combined with pembrolizumab (αPD-1)	Lang et al., 2018^94^	IT	glioma	R-IV	1	37	0	tumor reductions in 18/25 of patients 5/25 patients had a tumor reduction >95% and survival > 3 years	12/37 patients assed for IT changes (and not for clinical outcome). After 2 weeks from DNX-2401 administration: higher levels of CD8^+^ compared to CD4^+^ T cells.

**TLR ligands**									

Glycopyranosyl lipid A in stable-emulsion formulation (GLA-SE, TLR4) in combination with radiotherapy	Seo et al., 2023^95^	IT	STS	IV	1	12	0	all patients achieved local control with 1 CR	durable local response is associated with increases in TILs.
CM B305, a vaccine integrating a ZVEx-based lentiviral vector encoding for NY-ESO-1 (LV305) as the priming component and the TLR4 ligand (G305), administered in combination with atezolizumab (αPD-L1) (Arm A) vs. atezolizumab only (arm B)	Chawla et al., 2022^96^	IM (G305) + ID (CMB305)	SS + MLS	ADV-R-IV	2	89	arm A: 4 arm B: 4	mPFS: arm A. 2.6 vs. B. 1.6 months. mOS: 18 months for both arms, but OS of 36 months in the subset of patients with anti-NY-ESO-1 T cell immune response	arm A: higher rate of induced NY-ESO-1-specific T cell responses (53.8% vs. 15% of arm B) and induced NY-ESO-1-specific antibody responses (50% vs. 0%).
Imiquimod (TLR7)	Yoon et al., 2025^97^	topic (cream) on tumor area	OSCC	I-II	1	15	2	14/15 (93%) patients had a clinical tumor regression, with 9/15 major pathological response (of which 2 CR)	increases in IT Th cells, activated CD8^+^ T cells, and memory T cells (both CD4^+^ and CD8^+^) (compared to pre-treatment biopsies).
Imiquimod (TLR7) combined with radiotherapy with (Arm A) or without (Arm B) cyclophosphamide 1 week before study treatment vs. radiotherapy/chemotherapy only controls	Adams et al., 2025^98^	topic (cream) on tumor area	BrC	IV	1	31	4	2 CR + 2 PR imiquimod treated patients (arm A + B). local objective responses were observed in 19/24 (9 CR and 10 PR) areas treated with both imiquimod and radiotherapy compared to 5/24 (5 PR) lesions treated with only imiquimod	higher levels of immune infiltration in CR compared to the non-CR samples.
LHC165 (TLR7) alone (Arm A) or in combination with spartalizumab (αPD-1) (Arm B)	Curigliano et al., 2024^99^	IT	various	ADV	1/1b	45	1	best response: arm A. 1/21 PR, B. 2/24 PR, and 5/24 SD. mPFS: arm A. 1.7 vs. B. 1.8 months	responders (PR and SD) had higher levels of IT CD8, CD68, and PD-L1. These also increased more during treatment.
BDB001 (TLR 7/8)	Patel et al., 2020^100^	IV	various	ADV	1	36	3	6% durable PR, 56% SD DCR: 62%	BDB001 increased serum levels of IFNγ and IP-10, as well increased activation of DC and macrophages.
Vidutolimod (TLR9) combined with pembrolizumab (αPD-1)	Ribas et al., 2021^101^	IT	MEL	IV	1b	44	20	durable responses in 25% of patients (4 CR, 7 PR) regressions of both injected and non-injected lesions	responders had non-inflamed baseline tumors and induction of an IFNγ signature after treatment. Vidutolimod increased serum levels of IP-10.
Tilsotolimod (TLR9) combined with ipilimumab (αCTLA-4)	Haymaker et al., 2021^102^	IT	MEL	III-IV	1/2	62	34	for patients receiving the recommended dose (n:49): OOR = 22.4% (2 CR, 9 PR) 49% of patients had tumor regression, which was observed in both injected and non-injected lesions mPFS: 5.1 months mOS: 21.0 months	clinical response correlated to rapid induction of local IFNα gene signature, DC maturation, and T cell clonal expansion.
Vidutolimod (TLR9) combined with nivolumab (αPD-1)	Davar et al., 2024^103^	IT	MEL	III	2	31	0	55% major pathological response (Residual viable tumor 0–10%) with 14 CR, 3 PR. Other 3 patients had a PR (residual viable tumor < 50%). 1-year PFS: 77% (94% considering major responders)	major responses were associated with increased tumor-infiltrating CD8^+^ TILs and pDC.
SD-101 (TLR 9) combined with nivolumab (αPD-1)	Cohen et al., 2022^104^	IT	HNSCC	mostly IV	2	28	12	2 CR, 10 PR. OOR: 24% 9-month PFS: 19.0% 9-month OS: 64.7% ORR in PD-L1 low tumors 22.9% vs. high 26.7% responses of both injected and non-injected lesions	on therapy, SD-101 biopsies showed increases in immune gene expression, including CD8^+^ T, activated NK, Th1, and B cells. Changes were seen primarily in responders.
Pixatimod (TLR9) combined with nivolumab (αPD-1)	Lemech et al., 2023^105^	IV	PDAC + CRC (MSS)	III-IV	1	58	21	PDAC (n:18): no responders CRC (n:25 evaluable): 3 PR and 8 SD. ORR for CRC: 17%	clinical benefit associated with lower pan-immune-inflammation value, lower plasma IL-6, higher CXCL10, and increased CXCL10/IL-8 ratio.

(m)OS, (median) overall survival; (m)PFS, (median) progression-free survival; AdV, adenovirus; ADV, advanced; BCC, cutaneous basal cell carcinoma; BrC, breast cancer; cDC, conventional DC; CR, complete response; CTLA-4, cytotoxic T lymphocyte-associated protein 4; DC, dendritic cell; DCR, disease control rate; GM-CSF, granulocyte monocyte colony-stimulating factor; HNSCC, head and neck squamous cell carcinoma; IFN, interferon; IL, interleukin; i.p.: intraperitoneal; IT, intratumoral; i.v., intravenous; MEL, melanoma; NKT, natural killer T cell; OC, ovarian cancer; ORR, overall response rate; PD-(L)1, programmed death-(ligand)1; PDAC, pancreatic ductal adenocarcinoma; pDC, plasmacytoid DC; PR, partial response; R, recurrent; RCB, residual cancer burden index; RES, resectable; s.c., subcutaneous; SD, stable disease; STING, stimulator of interferon genes; STS, soft tissue sarcoma; Th, T helper; TILs, tumor-infiltrating lymphocytes; TLR, Toll-like receptor; TLS, tertiary lymphoid structures; Tregs, regulatory T cells; T-VEC, talimogene laherparepvec.

a of DC immunomodulatory treatment.

b related to DC immunomodulatory treatment.

### CD40 agonists

CD40 is a transmembrane cell surface glycoprotein of the tumor necrosis factor (TNF) superfamily, expressed on stromal and cancer cells, APCs, and B cells. CD40L, mainly expressed by Th cells, activates DCs via CD40; thus, they are optimally equipped to prime cytotoxic T lymphocytes (CTLs), providing them with “a license to kill.”^106^ CD40 agonists mimic this interaction, promoting DC maturation by upregulating MHC molecules, co-stimulatory molecules (e.g., CD80/CD86), and IL-12 production, fueling the expansion of tumor-specific CTLs.^107^ Within solid tumors, both pDCs and cDCs can express CD40, with the latter having a higher expression.^108^^,^^109^ CD40 agonists can also skew macrophages toward anti-tumoral phenotypes, reducing immune suppression on infiltrating CTLs.^110^ Despite promising preclinical results, first-generation CD40 agonists in clinical trials encountered challenges, including dose-limiting toxicities.^111^ Furthermore, immunoglobulin (Ig)G1 CD40 agonists (e.g., mitazalimab) rely on Fcγ receptor-mediated crosslinking for their activity and rapid systemic clearance. Therefore, the level of Fcγ receptor expression in the TME/TDLN is rate limiting.^112^ IgG2 CD40 agonists (e.g., selicrelumab) can overcome this by achieving receptor clustering independent of Fcγ receptor engagement.^113^ Bispecific forms of selicrelumab that specifically target DCs (via CD11c, DEC-205, or CLEC9A arms) have shown potent anti-tumor activity in preclinical models,^114^ suggesting that DC activation is not the cause of the observed dose-limiting toxicities. In the clinic, the tumor/stroma-targeting bispecific, fibroblast activation protein (FAP)-CD40, increased DC-LAMP^+^ DCs and reduced CLEC9A^+^ DCs (cDC1) within solid tumors, with the full results yet to be published.^78^

Advancements of CD40 agonists have also focused on understanding and overcoming resistance. Preclinical responsiveness was dependent on pre-treatment cDC1-primed CD8 T cells in the TME and post-treatment cDC2s and migDCs driving IL-12-mediated T cell responses.^115^ In mice, chemotherapy could sensitize αCD40-resistant tumors by inducing immunogenic tumor cell death, propagating DC migration and systemic anti-tumor immune responses.^116^ PDAC is highly immunosuppressive with limited tumor neoantigen expression; hence, utilizing immunotherapy is particularly challenging. When selicrelumab was administered with chemotherapy in resectable PDAC patients,^79^ an OS of 100% at 1 year was recorded, associated with enrichment of intratumoral T cells and mature DCs (DC-LAMP^+^). Similarly, mitazalimab delivery 7 days prior to chemotherapy (FOLFRINOX) in metastatic PDAC patients caused fluctuations in DC (CD11c^+^) levels, but overall expansion of CD38^+^ NKT cells, which was significantly associated with a reduction in tumor size.^80^ The mOS with this combination therapy was 14.3 months, 3 months longer than that observed in previous trials of FOLFRINOX alone.^117^ This study reported high rates of adverse events; however, a phase 3 trial is now being prepared to compare combination therapy vs. chemotherapy alone.^118^

Further CD40 agonist combinations are being investigated in ongoing early-phase clinical trials. For example, in breast cancer (BrC), the clinically approved anti-angiogenic agent bevacizumab could enhance intratumoral immune cell infiltration and overcome VEGF-mediated immune suppression.^119^ A recent phase I trial was completed combining selicrelumab with bevacizumab in patients with advanced solid tumours (NCT02665416). Follow-up trials are already underway combining selicrelumab, bevacizumab, and atezolizumab (αPD-L1) (NCT03424005). Additional CD40 agonists and ICI combinations were recently summarized elsewhere, with ICIs able to enhance the function of tumor-specific CD40-primed T cells in the TME.^79^^,^^120^ Overall, second-generation CD40 agonists, when combined with other forms of immunotherapy, demonstrate clinical potential, specifically in PDAC (both respectable and metastatic), which is notoriously difficult to treat.

### STING agonists

STING is broadly expressed by immune and non-immune cells and is a crucial protein involved in the cGAS-STING DNA-sensing pathway, responding to intracellular double-stranded DNA. When activated in DCs, STING induces canonical type I IFN responses, which can act in a positive feedback loop to increase DC activation, enhancing antigen presentation and subsequent stimulation of cytotoxic CD8 T cells, as well as activating NK cells and other immune effector cells.^121^ All human DC subsets display potent activation via STING, with the cDC2s response being the strongest.^4^ Currently, the main method to target STING in the TME is through cyclic dinucleotide (CDN) analogs delivered intratumorally to boost a locoregional type I IFN response. Although first-generation analogs demonstrated good safety, they had only modest efficacy, and responses were limited to known, injectable lesions.^122^^,^^123^ Low efficacy can, in part, be attributed to the fact that STING activation can also have pro-tumoral effects within the TME, as chronic exposure to STING can dampen T cell responses by inducing apoptosis and upregulating PD-L1 on DCs.^124^^,^^125^

STING agonists with improved chemical structures and delivery methods to enhance potency are currently under development and testing in early clinical trials.^122^^,^^123^ Novel CDN analogs have shown mixed results when combined with αPD-1, with better outcomes associated with increased CD8 T cell infiltration; however, DC monitoring data are not available.^81^^,^^82^ CDN-infused exosomes, such as ExoSTING,^126^ aim to make potent delivery more targeted, with a phase 1 trial recently completed (NCT04592484) and results pending publication. Engineered bacterial vectors that produce STING-activating molecules have also been explored, aiming to provide sustained and targeted STING activation within tumors. SYNB1891, a modified *E. coli*, was shown to be taken up by DCs and macrophages pre-clinically,^127^ with a phase 1 clinical trial inducing stable disease in patients refractory to prior PD-1/L1 antibodies.^83^ To target non-injectable tumors, there is currently an ongoing phase 1 trial using a second-generation STING agonist (BI 1703880) via systemic administration, combined with ezabenlimab (αPD-1) (NCT05471856).

While there have been advancements in developing novel STING agonists, they have often failed to progress past early clinical trials. Nevertheless, ongoing investigations aim to create a balance between potent STING activation in desired cell types and minimal off-target effects (systemic toxicity or pro-tumoral responses).

### Cytokines

Cytokines are powerful immunomodulators; hence, pro-inflammatory cytokines make attractive candidates to boost DCs anti-tumor functions. Early trials with cytokines used systemic administration, leading to unacceptable side effects, so most recent trials aim to overcome this or instead utilize local/intratumoral delivery.^128^ Cytokine treatment has advanced (pre-)clinically by engineering DC/tumor vaccines to express cytokines that promote DC recruitment and/or activation^129^^,^^130^ or by applying cytokine genes directly as genetic adjuvants, either encoded by DNA plasmids, mRNA molecules, or (oncolytic) viruses.^131^

Flt3 ligand (Flt3L) is a tyrosine kinase receptor ligand critical for cDC and pDC development, as evidenced by studies in *Flt3*- and *Flt3L*-deficient mice.^8^^,^^9^ Systemic Flt3L administration in preclinical cancer models increased anti-tumoral cDC1s, but clinical trials have so far failed to achieve similar responses and sufficient efficacy.^132^ To improve outcomes, engineered versions of Flt3L and combination with DC vaccines are being evaluated. GS-3583, a fusion protein comprising the extracellular domain of recombinant Flt3L fused to an engineered Fc region of human IgG4, was designed to overcome the short half-life of rFlt3L. This was recently administered i.v. in a phase 1b study with cDC expansion noted; however, it had to be terminated due to the treatment-related induction of a second primary malignancy,^84^ perhaps indicative of remaining systemic toxicity. In a recent phase 2 trial in resected melanoma, Flt3L (CDX-301) was administered subcutaneously prior to the DC vaccine anti-DEC-205-NY-ESO-1 (a fusion antibody targeting CD205) and Poly:IC.^85^ Flt3L augmented immune responses, including a mean cDC and pDC increase of 27.8-fold and 15-fold from baseline, respectively, associated with enhanced NK and T cells. Unfortunately, this trial did not include a non-vaccinated control arm, so clinical benefit was not assessed.

GM-CSF facilitates DC recruitment, maturation, migration, and IL-12 secretion.^10^^,^^133^ rhGM-CSF (sargramostim/Leukine) is Food and Drug Administration (FDA) approved for acute myeloid leukemia, used post-chemotherapy to reduce infection by promoting myeloid recovery. However, clinical trials have shown limited anti-tumor activity as a monotherapy,^128^ promoting efforts to combine GM-CSF with ICIs and DC vaccines. To target PDAC, allogeneic tumor cells virally transduced with GM-CSF (GVAX) were combined with (A) chemotherapy, (B) chemotherapy plus nivolumab (αPD-1), or (C) all of the previous plus urelumab (CD137 agonist).^86^ Arm C showed the greatest infiltration of CD8 T cells (DCs not mentioned), correlating with improved DFS of over 100% compared to arm A. Naxitamab (mAb against GD2) in combination with GM-CSF is approved in America (since 2020) and several other countries for patients with relapsed or refractory neuroblastoma in the bone who have demonstrated a PR, minor response, or stable disease to prior therapy. Unfortunately, trials that supported the clinical approval lacked immune monitoring, so DCs’ role in the anti-tumoral responses remains unclear.^87^ For the treatment of melanoma, many trials have employed GM-CSF as a DC vaccine adjuvant; however, the results are inconsistent.^128^

Currently, cytokine treatment yields variable clinical results, likely attributed to their potential dual roles in the TME, where they can also drive pro-tumoral myeloid subsets,^133^ hence complicating their therapeutic use and calling for further research to identify cancer types and patient subsets that would benefit from DC-modulating cytokine combination therapy.

### Oncolytic viruses

Oncolytic viruses (OVs) are either wild-type or genetically engineered viruses that only replicate in tumor cells, leading to selective tumor cell lysis and subsequent immune activation through the release of tumor-derived (*neo*)antigens and damage-associated molecular patterns (DAMPs).^134^^,^^135^ OVs can activate DCs directly, through interactions between OV-associated pathogen-associated molecular patterns (PAMPs) and pattern recognition receptors (PRRs) on DCs, or indirectly via DAMPs released from virus-induced necrotic cells, as well as through stimulatory signals from other cells that respond to PAMPs/DAMPs.^136^ Additionally, OVs have been engineered to enhance their DC-activating capacity by expressing cytokines (e.g., GM-CSF), aiming to localize immune activation to the tumor site, enhance efficacy, and minimize systemic toxicity. To date, three OVs have been approved for clinical use: Oncorine, an engineered adenovirus (AdV), approved in China for the treatment of nasopharyngeal carcinoma^137^; Talimogene laherparepvec (T-VEC), an engineered GM-CSF expressing oncolytic herpes simplex virus (oHSV), approved in the USA and Europe for the treatment of advanced melanoma^138^; and Delytact, an oHSV approved in Japan for the treatment of malignant gliomas (including glioblastoma).^139^ Other OVs currently under clinical development exploit various virus types, including HSV, AdV, measles, and vaccinia viruses, which have been reviewed recently by Lowenstein and colleagues.^140^

Most advancements have focused on DC-activating OVs, particularly GM-CSF encoding T-VEC, which induces DC attraction and maturation, resulting in enhanced T cell priming.^136^ A recent systematic review examined clinical efficacy in 779 patients with advanced melanoma, where intervention led to an ORR of 62.2% and a durable response rate of 41.87%.^141^ Similarly, encouraging results were obtained in a phase 2 trial of cutaneous basal cell carcinoma, achieving the primary endpoint of tumor resectability after enrolling half of the planned number of patients.^88^ ORR was 55.6%, with an OS rate of 100% at 6 months, with increased CD8 T cells, B cells, and CD68^+^ myeloid cells after treatment; however, no difference was observed in DC abundance between patients with a pathological CR or non-complete response. Recent developments have also focused on ICI combination therapy that can benefit from the pro-inflammatory shift in the TME. In a systematic review analyzing 15 studies of OV therapy combined with ICI or chemotherapy in solid cancer patients, ICI combination induced a 39% ORR, compared to 26% for OV monotherapy.^142^ T-VEC with ipilimumab (αCTLA-4) enhanced clinical responses in advanced melanoma patients compared to ipilimumab monotherapy,^89^ whereas T-VEC with pembrolizumab (αPD-1) in unresectable/metastatic melanoma patients induced limited benefits compared to pembrolizumab monotherapy^90^ but improved PFS in patients with αPD-1 therapy resistance, especially when administered at an earlier disease stage.^91^ Immune monitoring was not conducted in these trials, so no information is provided on how the treatment affected DCs. Additionally, T-VEC with αPD-(L)1 had limited clinical benefit in trials of head and neck squamous cell carcinoma, triple-negative BrC, and hepatocellular carcinoma,^92^^,^^93^ suggesting that not only is the choice of ICI as a combination therapy important but also is the patient cohort.

Overall, strong evidence is accumulating to support the clinical use of OVs. Indeed, classical OVs show remarkable results in some patient groups, showcased by the oncolytic AdV DNX-2401 inducing long-term (>3 years) clinical responses in a subgroup of recurrent glioma patients after only a single intratumoral dose.^94^ T-VECs use in certain tumor types (e.g., cutaneous basal cell carcinoma) or patient subgroups (e.g., acquired resistance to PD-1) has shown encouraging results that warrant further investigation. In terms of using OV as a DC-modulatory therapy, clinical information on the effect of OVs on DCs is still lacking, and DC-targeting OVs currently yield limited additional efficacy, with further developments needed to enhance their clinical application.^143^

### TLR ligands

TLRs are a family of PRRs expressed by immune and non-immune cells, including DCs. Ten human TLRs have been identified: six on the cell surface (1, 2, 4, 5, 6, and 10) and four localized to endosomes (3, 7, 8, and 9).^144^ Currently, only two TLR agonists are FDA approved for cancer: Bacillus Calmette-Guerin (acting as a TLR2/4 and TLR9 agonist) against non-muscle-invasive bladder cancer and imiquimod, a TLR7 agonist for superficial basal cell carcinoma.^144^ Moreover, the TLR4 agonist Picibanil is approved in Japan for use with chemotherapy for gastric and lung cancer. Currently, the major focus of ongoing clinical development lies in combining TLR agonists with ICIs and other immunotherapies,^144^^,^^145^ reflecting the trend to preferentially explore synergistic strategies that enhance multiple steps in the cancer-immunity cycle.^6^^,^^19^ TLR4, predominantly expressed by myeloid cells, can induce pro-inflammatory cytokine secretion (IL-1β, IL-6, and TNF), promoting DC maturation and migration, and Th1 lymphocyte skewing.^146^ Among surface TLRs, TLR4 is the most frequently targeted in trials, investigated as a vaccine adjuvant or as part of a combination therapy. Recent trials, such as combining a TLR4 agonist glycopyranosyl lipid A in stable-emulsion formulation with radiotherapy^95^ or using the TLR4 ligand G305 as a priming component for an NY-ESO-1 vaccine (CMB305), combined with atezolizumab (αPD-L1),^96^ showed clinical responses (Table 2); however, DC monitoring was not conducted so it is unclear if the clinical benefit was through DC modulation.

Endosomal TLRs, particularly TLR7/8/9, have been the focus of many recent clinical trials.^144^ TLR7 is predominantly expressed by pDCs, while TLR8 is more abundant on cDCs; both are also expressed by monocytes, macrophages, and T cells. TLR7 activation triggers type I IFN responses through IFN-regulatory factor 7, whereas TLR8 activation promotes pro-inflammatory cytokine release, such as IL-12, via activation of nuclear factor kappa-light-chain-enhancer of activated B cells.^146^ Recent studies have aimed to expand the use of the approved TLR7 agonist imiquimod to more patient groups. Previous patient studies have shown that pDCs migrate to imiquimod application sites and produce type I IFNs, promoting tumor cell killing. However, it should also be noted that TLR-activated pDCs can also induce Tregs in the TME, associated with poor prognosis.^147^ When given as a neoadjuvant treatment to early-stage oral squamous cell carcinoma patients in a phase 1 trial, it led to 93% clinical tumor regression, including two patients with CR.^97^ DCs were not investigated in this trial, but a 709% increase in CTLs was recorded, highlighting that best responses of TME modulation may come from early intervention. Encouraging results were also observed in metastatic BrC patients when imiquimod was combined with radiotherapy, leading to responses in 19/24 areas treated, with nine of those demonstrating CR, compared to 5/24 PR for imiquimod alone.^98^ CRs displayed increased DCs compared to non-responders. In contrast, the αPD-1 combination induced only limited benefits compared to TLR7 agonist monotherapy.^99^ Dual TLR7/TLR8 agonists also show promise, as observed after systemic administration of BDB001 in a phase 1 trial of patients with advanced solid tumors, inducing a disease control rate (DCR) of 62%, associated with DC and macrophage activation and increased IFNγ in serum. Interestingly, patients previously progressing on αPD-L1 therapy in the 6 months prior to BDB001 treatment had better responses than patients progressing on prior chemotherapy.^100^

An alternative mechanism to target pDCs is through TLR9 agonists, which promote pDC activation and rapid type I IFN release. The latter induces cDC maturation and enhances their cross-priming ability.^146^ Two complementary studies have shown encouraging results combining intratumoral TLR9 (vidutolimob or tilsotolimod) with systemic ICI in melanoma.^101^^,^^102^ Vidutolimob-pembrolizumab (αPD-1) achieved a 25% DCR,^101^ and tilsotolimod-ipilimumab (αCTLA-4) resulted in a 22.4% response rate with an additional 49% SD.^102^ In both trials, tumors regressed in both injected lesions and distant, non-injected sites, indicating systemic immune activation, which correlated with clinical responses (including IFN-related gene expression). Vidutolimod-pembrolizumab responders exhibited increased DC maturation and T cell clonal expansion, whereas tilsotolimod-ipilimumab responders showed increased CD8^+^ T cell infiltration. Interestingly, vidutolimob-pembrolizumab efficacy was more associated with pDC abundance than with T cell infiltration. Expanding to a phase 2 trial, intratumoral vidutolimod-nivolumab therapy led to a 55% major pathological response rate, associated with post-treatment CD8 T cell and pDC infiltration.^103^ Beyond melanoma, TLR9 agonist-ICI combinations are advancing in other cancers. In a phase 2 trial in head and neck squamous cell carcinoma, responses to the TLR9 agonist SD-101 occurred in both PD-L1 high and low tumors,^104^ suggesting benefits for patients who do not respond to ICIs due to low PD-L1 levels. Additionally, in microsatellite stable colorectal cancer (MSS-CRC), a notoriously cold tumor type, promising results were reported for combined TRL9 agonist/PD-1 blockade therapy, with 12% achieving PR and 44% DCR, with clinical outcomes correlating with increased DCs and T cells; however, no effects were observed in PDAC.^105^ Importantly, while the rate of adverse events for the TLR9-ICIs combination was found to be acceptable with local TLR9 administration,^102^^,^^103^^,^^148^ systemic administration led to grade 5 AE.^105^

Based on recent and ongoing clinical trials, combining TLR agonists with radiotherapy or ICI shows promise in tumor types that are often difficult to induce responses in.^95^^,^^98^ Clinical benefits are often associated with DC or T cell activation, suggesting that immune monitoring may help identify early responders. Therefore, the therapeutic potential of TLR agonists supports their continued development, particularly with immunomodulatory combinations and in early disease settings.

## Concluding remarks: Challenges and future directions for DC-based immunotherapy

DC-based cancer immunotherapy harnesses DCs’ unique capacity to bridge innate and adaptive immunity, thereby cyclically priming tumor-specific effector T cells and stimulating cancer cell cytotoxicity. This review discusses recent advancements in both anti-tumoral therapeutic vaccination and DC-modulatory therapies. In recent years, the majority of DC immunotherapy clinical trials have concentrated on anti-tumoral therapeutic vaccination, utilizing both *ex vivo*-generated DCs and non-cell-based vaccines (e.g., mRNA-based), with successful outcomes often correlating with the use of appropriate agonists, a low tumor burden either due to early stage or therapy induced, (controversially) a cold TME at baseline, well-timed combination therapies, and a measurable induction of T cell responses.

In terms of DC-modulatory therapies, most recent trials have utilized TLR ligands and OVs, whereas CD40 agonists, STING ligands, and cytokine therapies have seen comparatively fewer clinical studies. Among these, endosomal TLR targeting has driven effective anti-tumoral DC responses in the TME, showing more favorable outcomes when combined with radiotherapy or ICIs.^98^^,^^101^^,^^102^ Despite this, most phase 3 trials have utilized TLR ligands as vaccine adjuvants rather than primary cancer therapies, likely reflecting the ongoing concerns of immune overactivation and emphasizing the need for biomarkers to identify responsive patient cohorts. Fcγ-independent CD40 agonists are also progressing to phase 3 trials,^118^ underscoring the growing confidence in this therapy. Moreover, bispecific CD40 agonists are being explored to enhance their specificity within the TME.^78^ In contrast, DC-targeting OVs, STING agonists, and cytokine therapies have shown mixed clinical results. DC-specific OVs provide limited benefits over non-targeted OVs, and STING trials often lack mechanistic insight due to minimal DC monitoring.^81^^,^^82^ While advances in STING delivery (e.g., engineered exosomes) may improve delivery specificity, these approaches remain in the early clinical validation.^126^ Cytokine therapies like Flt3 and GM-CSF have largely moved away from monotherapies; however, in combination with DC vaccination,^84^^,^^85^^,^^128^ they have produced only minimal positive clinical outcomes directly attributable to DC modulation. This may be attributed to the complicated and sometimes dual roles of cytokines in the TME.^133^

Notably, most clinical studies discussed are recent phase 1 or 2. While this reflects the most cutting-edge clinical advances, it also limits broader conclusions due to inconsistent trial design, limited immunomonitoring, and heterogeneous patient populations, further complicating interpretation. Nevertheless, some trends in clinically relevant variables can be identified. As shown for anti-tumoral therapeutic vaccination, reduced immune suppression, either due to early clinical stage or therapy induced, and a sustained T cell response are key conditions for therapeutic success.^1^ Not surprisingly, these characteristics also appear applicable to DC-modulatory therapy. Indeed, while most studies focused on advanced malignancies, remarkable responses were achieved with DC modulation in earlier disease stages with TLR7 and CD40 agonists.^79^^,^^97^ Moreover, clinical responses were often associated with local and/or systemic T cell activation. Furthermore, immune-cold tumors also encouraged successful therapeutic outcomes for both DC vaccines, where ovarian tumors responded even better to an autologous DC vaccine than hot tumors,^70^ and CD40 and TLR agonists, which induced clinical responses in notoriously cold tumors such as glioblastoma, PDAC, and MSS-CRC.^66^^,^^67^^,^^80^^,^^105^ This may be attributed to a lower immunosuppressive state in cold tumors compared to highly infiltrated tumors, where suppressive compensatory mechanisms might develop in response to the increased effector cell presence, ultimately limiting the efficacy of the vaccine. Also, it makes sense that these cold tumor types in particular would benefit from T cell priming therapies, perhaps more so than hot tumors with pre-existent anti-tumor T cell responses and an inherently more favorable prognosis.^149^

Beyond immune-cold tumors, novel DC-based immunotherapies also show potential for patients with αPD-1 resistance. Clinical responses to TLR9 for head and neck squamous cell carcinoma occurred in both PD-L1 high and low tumors,^104^ suggesting a benefit for patients who do not respond to ICIs due to low PD-L1 levels. Additionally, T-VEC with pembrolizumab (αPD-1) in advanced melanoma patients provided benefits to αPD-1 therapy resistance patients^91^; however, this was specific to an earlier disease setting. Many trials have attempted to combine DC-based immunotherapy with ICIs to counteract immune suppression in the TME and induce *de novo* T cell priming, thereby facilitating more effective immune checkpoint blockade. However, variability in outcomes may be due to multiple factors, including tumor stage and load, tumor types and their differential inherent immunogenicity, the DC subset targeted, delivery routes, anatomical compartment targeted, and differences in immune suppressive conditions and pathways involved.

In conclusion, DC-based immunotherapies, particularly vaccination, TLR ligands, and CD40 agonists, have demonstrated the ability to induce DC-specific anti-tumor immune responses associated with improved survival across a range of difficult to treat tumor types, including immune-cold and those resistant to standard immunotherapies. Currently, most advances have only made it into early-stage trials, as both vaccination and DC-modulation strategies face several common challenges in optimizing delivery platforms and overcoming immune suppressive conditions in late-stage TMEs. In relation to the former, dosing and administration require optimization; for example, only a small percentage of injected MoDC-based vaccine DCs reach the LN.^150^ For DC-modulatory therapy, improving systemic potency while maintaining reduced systemic inflammation remains challenging. Improving DC-specific targeting has been presented as a potential solution, e.g., using bispecific antibodies that target antigens and/or immune modulatory agents directly to DCs in the TME and/or TDLN.^78^^,^^114^ Overall, continued refinement of delivery methods, combinatorial strategies, and deepening our understanding of the mechanisms that underlie DC activation versus suppression in the TME will allow for further advances of current and novel DC therapies.

### Data and code availability

No new data were created or analyzed in this study. Data sharing is not applicable to this article.

## Author contributions

Conceptualization: G.C., E.C.T., T.D.d.G., and Y.v.K.; investigation: G.C. and E.C.T.; writing – original draft preparation: G.C. and E.C.T.; writing – review and editing: G.C., E.C.T., T.D.d.G., and Y.v.K.; supervision: T.D.d.G. and Y.v.K.; all authors have read and agreed to the published version of the manuscript. This work was funded by Oncode Accelerator, a Dutch National Growth Fund project under grant number NGFOP2201.

## Declaration of interests

The authors declare no competing interests.
